# Understanding Medical Distrust Among African American/Black and Latino Persons Living With HIV With Sub-Optimal Engagement Along the HIV Care Continuum: A Machine Learning Approach

**DOI:** 10.1177/21582440211061314

**Published:** 2021-12-01

**Authors:** Ning He, Charles M. Cleland, Marya Gwadz, Dawa Sherpa, Amanda S. Ritchie, Belkis Y. Martinez, Linda M. Collins

**Affiliations:** 1 New York University Silver School of Social Work, USA; 2 New York University School of Medicine, USA; 3 Fordham University Graduate School of Education, New York, NY, USA; 4 New York University School of Global Public Health, USA

**Keywords:** medical mistrust, medical distrust, HIV care continuum, machine learning, random forest, racial/ethnic disparities

## Abstract

Medical distrust is a potent barrier to participation in HIV care and medication use among African American/Black and Latino (AABL) persons living with HIV (PLWH). However, little is known about sociodemographic and risk factors associated with distrust. We recruited adult AABL PLWH from low socio-economic status backgrounds with insufficient engagement in HIV care (*N* = 512). Participants completed structured assessments on three types of distrust (of health care providers, health care systems, and counter-narratives), HIV history, and mental health. We used a type of machine learning called random forest to explore predictors of trust. On average, participants were 47 years old (*SD* = 11 years), diagnosed with HIV 18 years prior (*SD* = 9 years), and mainly male (64%) and African American/Black (69%). Depression and age were the most important predictors of trust. Among those with elevated depressive symptoms, younger participants had less trust than older, while among those without depression, trust was greater across all ages. The present study adds nuance to the literature on medical distrust among AABL PLWH and identifies junctures where interventions to build trust are needed most.

## Introduction

After a diagnosis of HIV infection, persons living with HIV (PLWH) are encouraged to link to HIV primary care, engage in HIV primary care regularly, initiate HIV antiretroviral therapy (ART), maintain high levels of adherence to ART to achieve suppressed HIV viral load, and sustain suppressed HIV viral load over time, a series of steps called the HIV care continuum (Mugavero et al., 2013). In the United States, individuals from African American or Black and Latino (AABL) racial/ethnic backgrounds are substantially over-represented among the population of PLWH in comparison to their proportions in the general population (Centers for Disease Control and Prevention, 2018; Prejean et al., 2011; Xia, Braunstein et al., 2017). Moreover, serious racial/ethnic disparities persist in rates of engagement along the HIV care continuum (Crepaz et al., 2018; Dale et al., 2016). Of concern, AABL PLWH are less likely to achieve and sustain HIV viral suppression compared to their White peers (Crepaz et al., 2018). Yet, HIV viral suppression is vital for the optimal health and well-being of PLWH and also to prevent the forward transmission of HIV to others (Saha et al., 2010).

These significant HIV-related racial/ethnic health disparities are driven by factors that operate simultaneously at structural, social, and individual levels of influence (Dasgupta et al., 2016; Eberhart et al., 2015). At the structural level of influence, chronic poverty creates complex tangible barriers to engagement in HIV care and sustained ART use such as challenges accessing high-quality HIV services and unstable or low-quality housing (Aidala et al., 2016; Colasanti et al., 2017; Freeman et al., 2020). Social-level barriers to engagement along the HIV care continuum include internalized stigma and insufficient social support (Freeman et al., 2020; Maragh-Bass et al., 2020; Rice et al., 2017). At the individual level of influence, barriers include fear of ART side effects, substance use problems, mental health concerns such as depression (Bogart et al., 2010) and post-traumatic stress disorder (PTSD), and medical distrust (Brincks et al., 2019; Freeman et al., 2017; Kalichman et al., 2016). Because much of the research on PLWH is conducted in HIV care settings, those poorly engaged along the HIV care continuum are understudied compared to their better-engaged peers (Gwadz et al., 2015; Hartzler et al., 2017; Miles et al., 2019; Mimiaga et al., 2013). The present study focuses on the population of AABL PLWH from low socioeconomic status (SES) backgrounds who are engaged along the HIV care continuum at sub-optimal rates and who evidence unsuppressed HIV viral load, and seeks to advance the literature on one important type of barrier to HIV care continuum engagement, medical distrust.

Medical distrust is common among AABL PLWH and encompasses distrust of health care providers and medical systems (Bogart et al., 2010; Sohler et al., 2007; Whetten et al., 2008). HIV-related conspiracy beliefs, which we refer to as “counter-narratives” in the present study, are another aspect of distrust; examples include beliefs that HIV/AIDS is a form genocide against Black people and that ART is toxic (Bogart & Thorburn, 2005; Bogart et al., 2010; Galvan et al., 2017; Mackenzie, 2011; Quinn et al., 2018; Saha et al., 2010; Whetten et al., 2008). We use the term counter-narrative rather than conspiracy theory in the present study, consistent with critical race theory (Delgado & Stefancic, 2017), which encourages the elicitation of the lived experiences and perspectives of AABL individuals as a means of understanding the ways living in an unequal society is experienced (Delgado & Stefancic, 2017; Freeman et al., 2017). Further, the term counter-narrative highlights that such beliefs are not necessarily invalid, as they are shaped by, and grounded in, history and present-day reality, even if current evidence suggests in some cases, they are not entirely factually accurate. There is a consensus that experiences of medical distrust among AABL populations are at least partly rooted in past maltreatment, exploitation, and abuses of AABL populations at the hands of the United States federal government, health care system, and medical research establishment (Jaiswal, 2019; Scharff et al., 2010; Washington, 2006). In fact, incidents of exploitation of AABL populations in medical research date back over centuries (Scharff et al., 2010; Washington, 2006), the most infamous example of which is the Tuskegee Study of Untreated Syphilis in the Negro Male, commonly referred to as the Tuskegee Syphilis Study (Dale et al., 2016; Washington, 2006). Yet, as recently as the 1990s, unethical medical research involving AABL individuals has been conducted by academic and medical institutions (Scharff et al., 2010). While medical distrust may stem from these types of historical events, there is also a consensus that medical distrust among AABL populations is reinforced by present-day structural racism, inequality in the larger society and health care system, and discriminatory events (Adams & Simoni, 2016; Jaiswal & Halkitis, 2019; Randolph et al., 2020; Scharff et al., 2010; Williamson et al., 2019).

Past research has found that medical distrust is a major barrier to HIV care continuum engagement among AABL PLWH. Brincks et al. (2019) found that higher levels of physician distrust are associated with longer lapses of time between HIV care visits, which, in turn, are associated with higher HIV viral load levels. Conversely, higher levels of provider trust are associated with better HIV outcomes, increased HIV-related clinic visits, fewer emergency room visits, and better mental and physical health (Whetten et al., 2008). Of concern, medical distrust is related to lower continuous medication adherence over time (Bogart et al., 2010; Dale et al., 2016; Eaton et al., 2015; Thrasher et al., 2008). Medical distrust is also associated with poor mental health outcomes among AABL PLWH (Cunningham et al., 2007; Relf et al., 2019; Thrasher et al., 2008). Compared to their peers not living with HIV, PLWH are more likely to experience mental health symptoms such as depression and PTSD (Cohen et al., 2001; Cunningham et al., 2007; Safren et al., 2003), and these are associated with poor engagement along the HIV care continuum (Cunningham et al., 2007; Mutchler et al., 2019; Relf et al., 2019; Thrasher et al., 2008). Further, distrust of providers can impede HIV care continuum engagement in the context of substance use (Gwadz et al., 2016; Hartzler et al., 2017; Mimiaga et al., 2013). Substance use is prevalent among AABL PLWH at both non-hazardous and hazardous levels, but PLWH who use substances commonly stop ART or reduce ART doses to minimize the chances of potentially harmful interactions between HIV medication and substances (Gwadz et al., 2016; Hartzler et al., 2017; Mimiaga et al., 2013). Yet, low levels of trust in providers prevents PLWH from disclosing substance use in clinical encounters (Gwadz et al., 2016). Sociodemographic and background characteristics related to higher levels of medical distrust include being male, gay, lesbian, bisexual or queer sexual orientation, transgender or gender expansive gender identity, lower education levels, unemployment, incarceration history, and lower income (Bogart & Thorburn, 2005; Bogart et al., 2016; Zhang et al., 2020). Additionally, in a study of Black sexual minority men living with HIV conducted by Mutchler et al. (2019), the associations between trust in healthcare and higher ART adherence rates were greater for older than younger men.

Thus, medical distrust is an important impediment to optimal physical and mental health among AABL PLWH and serves as a barrier to engagement along the HIV care continuum. However, little is known about the factors associated with medical distrust in this subpopulation of AABL PLWH that is understudied because they are poorly engaged in HIV care settings where research is commonly conducted (Anderson et al., 2020), or about the relative importance of these factors and how they may interact with each other. To address these gaps in the literature, we used a machine learning approach (Liaw & Wiener, 2002) to explore associations among medical distrust and sociodemographic characteristics, background risk factors prevalent in the population (homelessness, incarceration, and indications of extreme poverty; Cargill & Momplaisir, 2021), substance use patterns, mental health symptoms, as well as potential interactions among these factors. Machine learning, as an approach to data analysis, is a collection of computer-intensive methods capable of yielding new insights with less reliance than classic statistical methods on prior understanding and explicit hypotheses (Efron & Hastie, 2016). Past research focused on relationships between medical distrust and HIV medication adherence have used a range of statistical analytic strategies such as logistic regression, multivariable regression, and generalized estimating equations (Dale et al., 2016; Eberhart et al., 2015; Hartzler et al., 2017; Mimiaga et al., 2013; Mutchler et al., 2019; Saha et al., 2010). The majority of these past studies have taken a hypothesis testing approach. While the hypothesis testing approach is valuable, we used a machine learning approach in the present study because we believe there are important interactions among factors determining distrust, but we lacked sufficient prior information and theory to specify those interactions and hypotheses about how they work. In instances such as these, the machine learning approach is useful because it excels at knowledge discovery (Efron & Hastie, 2016).

## Methods

### Design and the Larger Context

The present cross-sectional study used baseline data from a larger study. Participants (*N* = 512) were recruited from 2017 to 2019 in New York, NY as part of a research project focused on AABL PLWH from low-SES backgrounds who were not engaged in HIV primary care at recommended levels, had suboptimal adherence to ART, and evidenced unsuppressed HIV viral load (Gwadz et al., 2017). New York City is a location with more than 120,000 PLWH, the majority from AABL backgrounds (75%) (New York State Department of Health, 2015). HIV primary care and ART are available to PLWH in New York City at low or no cost (New York State Department of Health, 2015). Yet, racial/ethnic disparities in engagement along the HIV care continuum in New York City are similar to national patterns; AABL PLWH are over-represented in the population of PLWH and show the lowest rates of engagement in HIV care, ART uptake, and HIV viral suppression of any racial/ethnic groups (Xia, Robbins et al., 2017). The present study was approved by the Institutional Review Board of the New York University Grossman School of Medicine and informed consent was provided by participants for study activities.

### Eligibility Criteria

The inclusion criteria for the larger study were (1) age 18–65 years; (2) African American/Black race and/or Latino ethnicity; (3) diagnosed with HIV at least 6 months prior; (4) lack of engagement in HIV care at recommended levels (i.e., less than 1 visit every 4 months in the past year, or two or more missed visits without prior cancelation in the past year, as missed visits are independently associated with mortality (Mugavero et al., 2014); (5) ART adherence of less than half of prescribed doses in the past 6 weeks (by self-report) and unsuppressed HIV viral load based on a laboratory report; (6) residence in the New York City metropolitan area; and (7) ability to participate in research activities in English or Spanish. The participants were found eligible for the larger study if they met all the inclusion criteria.

### Recruitment and Procedures

We recruited participants using a multi-pronged sampling strategy comprised of peer-to-peer recruitment, recruitment by study staff members in HIV service settings, and advertisements placed in a free local newspaper. Potential participants contacted the study directly by phone, and usually with a coded recruitment coupon provided to them by a peer recruiter. After providing verbal informed consent, participants engaged in a brief screening interview. Those found preliminarily eligible at this stage proceeded to a second screening interview, where participants provided signed informed consent, presented confirmation of HIV status, and were escorted by a study staff member to a local commercial laboratory for HIV viral load testing. They were compensated $15 for the screening interview and $15 for completing the blood specimen draw, along with funds for local round-trip public transportation. HIV viral load results were available from the laboratory within 4 days. Those found eligible for the larger study based on meeting the HIV viral load criterion were invited to enroll in the study. After providing signed informed consent for enrollment, participants completed a structured baseline assessment battery lasting 60 to 90 minutes conducted on a cloud-based platform for data capture designed for clinical research called REDCap (Harris et al., 2009, 2019). The assessment battery was comprised of computer-assisted personal interviewing and audio computer-assisted self-interviewing formats. Individuals were compensated $25 for the baseline assessment, along with funds for round-trip local transportation.

### Measures

#### Demographic/background variables, substance use and mental health.

Demographic and background variables included age (in years), gender identity (cisgender male, cisgender female, and transgender), sexual orientation (heterosexual/straight, homosexual/gay/lesbian/queer/down-low, bisexual, and other) and race/ethnicity (African American/Black or Latino/Hispanic) (Chandler et al., 2015). Education level was coded as the receipt of a high school diploma or equivalent or above (yes/no). Current employment in a full-time or part-time job in the past 3 months was coded as yes/no. Incarceration history was assessed by asking whether participants had ever been held in a detention center, jail, or prison for more than 24 hours. If yes, participants were asked about incarceration in the past 6 months (Chandler et al., 2015). This was coded into a three-level variable: never incarcerated, past incarceration but not recent (i.e., incarcerated more than 6 months ago), and recent incarceration (within the past 6 months). Homelessness history was assessed by asking about participants’ current living arrangements, including temporary or unstable housing, and whether participants had been out-of-home in their lifetimes and recently. Data were coded as a four-level variable: currently homeless/unstably housed (yes/no), homeless in the past year but not currently (yes/no), homeless over the lifetime but not in the past year (yes/no), and never homeless (yes/no) (Chandler et al., 2015). To assess extreme poverty, we asked participants “During the past 12 months, how often did you run out of money for basic necessities like rent, utilities or food?” on a five-point scale ranging from never to daily (Chandler et al., 2015). We created a dichotomous variable indicating whether participants ran out of funds for necessities less than monthly or monthly or more. Participants answered the question, “How old were you when you got your first HIV-positive test result?” recoded as the number of years since HIV diagnosis (Hays et al., 1998). We assessed four indicators of substance use patterns from the WHO ASSIST instrument (WHO ASSIST Working Group, 2002) including risk category guided by pre-established algorithms (low-, moderate-, or high-risk use) for tobacco, alcohol, and cannabis and the highest risk category across nine other types of drugs (cocaine, hallucinogens, inhalants, methamphetamine, prescription opioids, prescription stimulants, street opioids, sedatives, and any other drugs). Symptoms of depression were measured using the 9-item Patient Health Questionnaire Depression Screen (Kroenke et al., 2001). Scores ranged from 0 to 27 and Cronbach’s α = .89. Participants completed the four-item primary care PTSD Screen (PC-PTSD) and those who answered yes to three or more items were coded as “likely PTSD” (Prins et al., 2003).

#### Primary outcomes: Medical trust.

We assessed three forms of medical trust: trust in health care providers, trust in the health care system, and counter-narrative beliefs. The three medical trust scales were administered in the audio computer-assisted self-interview format. We calculated a Percent of the Maximum Possible (POMP) score for each of the three scales. Counter-narrative beliefs were re-coded into a trust scale because the other two measures assessed trust (not distrust). In POMP, the score assigned is a percentage, reflecting the individual’s position on a 0 to 100 scale as a percent of the maximum possible score achievable on the scale (Cohen et al., 1999).

Trust in health care providers was assessed with an 11-item scale developed by Anderson and Dedrick (1990) to assess interpersonal trust in patient-physician relationships. Items (e.g., “I doubt that my health care provider really cares about me as a person,” “my health care provider is usually considerate of my needs and puts them first,” and “I trust my health care provider so much that I always try to follow his or her advice”) were coded on a five-point scale from strongly disagree to strongly agree. Items were reverse coded as appropriate and summed such that higher scores indicated more trust in medical providers (range 0–44; Cronbach’s α = .88) (Anderson & Dedrick, 1990).

Trust in the health care system was assessed with the Health Care System Distrust Scale (Hall et al., 2006; Rose et al., 2004; Shea et al., 2008). Participants reported their agreement with nine statements (e.g., “the health care system does its best to make patients’ health better,” “the health care system covers up its mistakes,” and “patients receive high-quality medical care from the health care system”) on a five-point scale from disagree strongly to agree strongly. Items were reverse coded as appropriate and scores ranged from 9 to 45, where higher scores indicated greater trust in the health care system (Cronbach’s α = .86).

Participants completed the HIV Conspiracy Beliefs scale (to assess counter-narrative beliefs), on which they reported their agreement with 10 items (e.g., “HIV is a manmade virus,” “people who take antiretroviral medications for HIV are human guinea pigs for the government,” “there is a cure for AIDS but it is being withheld from the poor,” and “the medicine that doctors prescribe to treat HIV is poison”) on a five-point scale from disagree strongly to agree strongly (Bogart et al., 2016). Prior to reverse-coding the measure, scores ranged from 10 to 50, where higher scores indicated greater endorsement of counter-narratives (Cronbach’s α = .87).

We also calculated an overall medical trust score. Because the three components of medical trust or distrust were correlated with each other (*r* = .43 between trust in provider and trust in healthcare system, *r* = −.42 between trust in healthcare system and counter-narrative beliefs, and *r* = −.26 between counter-narrative beliefs and trust in provider), we combined the three types of medical trust into one variable. Similar to the individual scales, the single variable was created by calculating a POMP score. Each component of overall medical trust was scored as percentage of maximum possible by subtracting the scale minimum from the respondents score and then dividing by the scale range. For counter-narrative beliefs, the percentage of maximum possible was subtracted from 100 so higher values would indicate greater trust. Further, we conducted both principal components analysis and confirmatory factor analysis to provide support for combining the three scales into a single POMP variable. In both principal components and confirmatory factor analysis, all three scales had substantial loadings on a single component/factor, and the POMP score was highly correlated with the component (*r* > .99) and factor (*r* = .94). Analyses were carried out with the three trust scales individually and the overall trust variable.

### Data Analysis

The present study used the random forest method, a type of machine or ensemble learning method in which many decision tree models are built, and their results are aggregated (Breiman, 2001; Pan et al., 2017). A regression tree can be constructed by considering every value of every variable (or feature) and then checking across all values for the single cut-point that will explain the most variability in the outcome in a regression context (Breiman, 2001). To deal with the risk of overfitting and to reduce the impact of correlation among potential predictors, Breiman (2001) introduced random forest as an improvement over single-tree models. The random forest procedure first selects the number of trees to build, and generates a separate bootstrap sample (with replacement) of the original data for each tree. For each split in the tree, the procedure randomly selects a subset of the predictors and then identifies the best split (e.g., the one minimizing within-partition variance) among the randomly selected subset of predictors. Each tree in the forest can be used to generate a prediction for a new sample, and these predictions are averaged to give the forest’s prediction. Since the algorithm randomly selects individuals for each tree and predictors at each split, tree correlation will necessarily be reduced. Individuals not included in a particular bootstrap sample are referred to as “out of bag” (OOB) (Kuhn & Johnson, 2013). The OOB prediction error is a summary of discrepancies between predicted and observed values when only individuals not included in a tree are considered. This is a form of cross-validation for the predictive model. The random forest approach was used in the present study in part because it can handle predictors with relatively high levels of multicollinearity, whereas multivariate regression frequently has difficulty with multiple predictors and multicollinearity (Kuhn & Johnson, 2013). The random forest approach is robust and equipped to address outliers automatically.

In this study, we selected predictor variables based on the existing literature and built a forest of 500 trees using the R programming language (Liaw & Wiener, 2002). At each split, four predictors were randomly selected for consideration. Several criteria were considered when judging the relative importance of predictor variables. The increase in mean squared error (MSE) of prediction indicates how much prediction error increases when a variable was not included in the tree. *Number of nodes* counts the number of times the predictor was the basis of a split. For both indices, a higher value indicates higher relative importance in predicting the medical trust variable. We recoded missing values for incarceration history (nine cases) and extreme poverty (six cases) as “unavailable,” so all 512 participants were retained in the analysis sample.

## Results

### Description of the Sample

Participants were 47.0 years old, on average (*SD* = 10.7 years), as shown in Table 1. Most were assigned male sex at birth (70.1%), African American/Black (68.6%), 8% were transgender, and 28.3% were homosexual/gay/lesbian/queer/down-low and 13.1% were bisexual. The majority (70.1%) had a high school education or higher, but only 8.2% were employed. Almost half (46.9%) had been incarcerated in the past but not recently, and 10.2% had been recently incarcerated. Homelessness and unstable housing were common; only 10.5% had never been homeless in their lifetimes. Indications of extreme poverty at least monthly were prevalent (57.1%). On average, participants had been diagnosed with HIV 18.2 years prior (*SD* = 8.61 years). Almost all (94.7%) had attended at least one HIV care appointment in the past year. Approximately two-thirds (67.6%) scored in the moderate-risk range for tobacco products. A little less than half (45.5%) scored in the moderate- or high-risk range for alcohol use. Approximately half (49.8%) scored in the moderate-risk range for cannabis use. Approximately half (47.5%) scored in the moderate-risk range for the highest risk category for other substances. The average score on the PHQ-9 depression index was 8 (*SD* = 6.40) and 29.1% met the criteria for likely PTSD.

### Trust POMP Scores

The POMP scores for the three individual trust variables ranged from 0 to 100 as noted above. The average POMP score for trust in providers was 68.7 (*SD* = 19.2). The average POMP score for trust in the healthcare system was 56.0 (*SD* = 22.4). The average POMP score for lack of counter-narrative beliefs was 57.3 (*SD* = 21.7). The overall POMP medical trust score ranged from 7.5 to 100 (mean = 60.6, *SD* = 16.0).

### Random Forest Analysis

#### Out-of-bag prediction error of random forest.

The overall out of bag (OOB) prediction errors were 15.6 (indicated by the square root of the mean of squared residuals) for the overall medical trust score, 21.45 for counter-narrative beliefs, 19.14 for trust in providers, and 21.81 for trust in the healthcare system. Thus, on the 0 to 100 trust scale, the random forest prediction for out of sample cases was typically off by nearly 16 points for the overall trust score, 19 points for trust in providers, 21 points for trust in the healthcare system and for counter-narrative beliefs. Approximately 5% of the variance in the overall trust score was accounted for by all 16 considered predictors, including interactions. Approximately 4% of the variance in system trust and 2% of the variance in counter-narrative beliefs was accounted for by the predictors. Variance accounted for in provider trust was essentially zero.

#### Variable importance and interactions.

Table 2 shows the 16 predictors according to variable importance criteria by outcome. Based on the increase in mean squared error (MSE) of prediction, age, depressive symptoms, tobacco risk, and years since HIV diagnosis were found to be the most important predictors for lack of counter-narrative beliefs. For trust in providers, age, depressive symptoms, likely PTSD, and years since HIV diagnosis were the most important predictors. For trust in healthcare system, depressive symptoms, likely PTSD, running out of money for necessities, and sexual orientation were the most important predictors. For the overall medical trust score, depressive symptoms, age, likely PTSD, and running out of money for necessities were the most important predictors. Notably, depressive symptoms were the first or second most important predictor for counter-narratives, healthcare system trust, and the overall trust variable.

**Figure 1.**
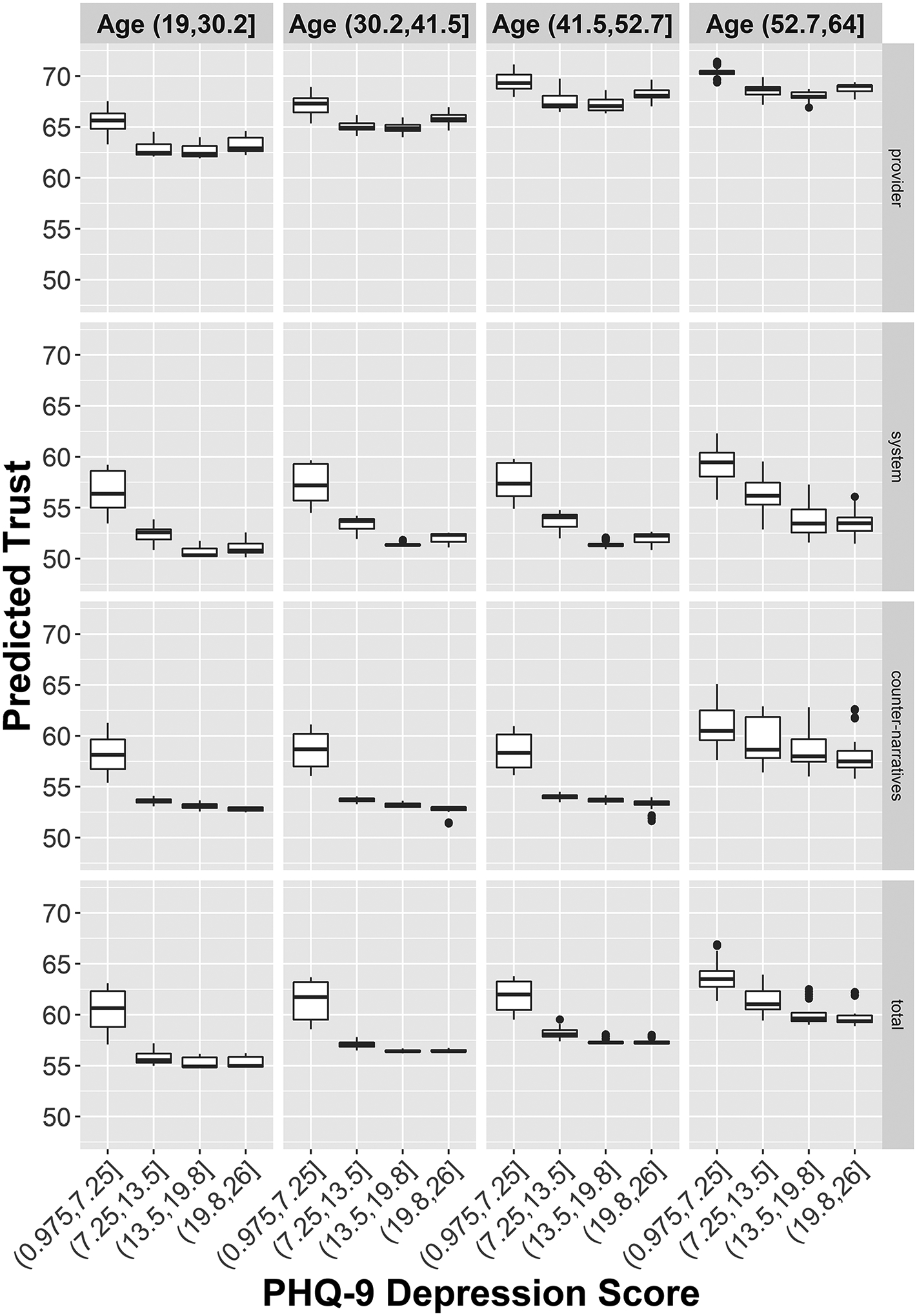
Marginal plot of the dependence of medical trust on the interaction of age and symptoms of depression.

Table 3 shows the 10 most important interactions for the three individual trust outcomes, calculated from the absolute value of the difference between their joint and additive importance. Interaction effects emerge naturally in regression trees. For example, if a tree was first split by sex assigned at birth, and then a subsequent split was made by incarceration history within the male branch of the tree, and this implies incarceration history is a predictor of the outcome among males but not females. An interaction between a variable and itself suggests a nonlinear association between a predictor and the outcome. Depressive symptoms by age was the most important interaction. Depressive symptoms were present in several other important two-way interactions as well. As shown in Figure 1, for all four outcomes, trust scores dropped steeply when depressive symptoms were elevated (PHQ-9 score ≥ 7.25). Generally, older age was associated with higher medical trust scores. Among those with elevated depressive symptoms, younger participants had lower levels of medical trust than older participants. Among those without depressive symptoms, medical trust scores were higher across all ages. Among the oldest PLWH in the sample, the decrease in medical trust scores with increasing depression was more gradual and leveled off at a higher level of trust when compared with younger PLWH.

We calculated marginal effects (Supplemental Table) to determine how other variables highest in importance were related to trust outcomes after taking other predictors into account (i.e., marginal effects integrating out the effects of other predictors). More years since HIV infection predicted a lack of counter-narrative beliefs. Being in the lowest risk category for tobacco use also predicted a lack of counter-narrative beliefs. Likely PTSD predicted less provider trust while more years since HIV diagnosis predicted more provider trust. Likely PTSD and running out of money for necessities more often were each associated with less system trust. Relative to homosexual/gay/lesbian/queer and heterosexual orientations, bisexual and other sexual orientations were associated with less system trust. Likely PTSD predicted less overall trust while more years since HIV diagnosis predicted more overall trust.

## Discussion

Numerous past studies indicate that medical distrust serves as a potent barrier to consistent participation along the HIV care continuum among AABL PLWH. The present study extends the past literature on medical distrust in several ways: We focus on an understudied population of PLWH, namely, AABL individuals insufficiently engaged along the HIV care continuum, examine three different types of medical trust, and use a machine learning approach to uncover factors associated with higher or lower levels of medical trust and their interactions. The random forest approach uncovered unexpected results, such as the interaction of age with depression, which we describe below, to thereby advance the literature on medical distrust.

We found different types of trust are moderately related to each other in the population of AABL PLWH, and examined them as separate domains and as a single multidimensional construct to understand patterns of prediction that are consistent and divergent across types of trust. Past studies with AABL PLWH in clinical settings have highlighted the multidimensional nature of medical distrust (e.g., PLWH can experience low levels of trust in the HIV health care system overall, but report substantial trust in their providers; Cunningham et al., 2007; Earl et al., 2013; Ford et al., 2013; Zhang et al., 2020). In the present study, trust in providers is higher than trust in the health care system and rejection of counter-narratives, consistent with these past findings. At the same time, the fact that these three variants of trust/distrust are associated with each other in this subpopulation of AABL PLWH suggests that when levels of overall medical distrust are high, such distrust can serve as a powerful barrier to HIV care continuum engagement. Yet, Earl et al. (2013) propose that medical distrust is not necessarily an undesirable attitude. In mental health settings, for instance, exhibiting distrust, caution, skepticism, or reservations about a provider or treatment plan can be considered a sign of resilience and/or a rational act of self-preservation in a society where racism is prevalent (Earl et al., 2013). Bogart et al. (2021) frame medical distrust among AABL PLWH as a survival mechanism that fosters coping with discrimination and helps prevent future mistreatment. While medical distrust certainly complicates HIV care continuum engagement, it does not preclude it. Understanding, eliciting, and addressing medical distrust generally, along with focusing on specific types of distrust, are critical to improving participation along the HIV care continuum, and thus health and wellbeing, among AABL PLWH, including efforts on the part of health care systems to earn trust, as we discuss in more detail below.

Of the factors examined, depressive symptoms have the strongest relationship to all types of medical trust/distrust, where medical trust drops sharply in the context of higher levels of depressive symptoms. There is a large literature indicating the relationship between depression and poor ART adherence (e.g., Sherr et al., 2011; Shi et al., 2019; Uthman et al., 2014). However, little is known about the association between depression and medical trust among PLWH. This paper fills the gap by exploring the importance of depressive symptoms as a predictor of medical trust. Our finding also suggests a specific mechanism by which depression might negatively impact ART adherence as well as engagement in HIV care generally. Given this is a cross-sectional study, greater distrust may contribute to greater depressive symptoms, higher levels of depressive symptoms may drive greater distrust, or depressive symptoms and distrust may have both similar underlying causes and reciprocal or reinforcing relationships with each other. One interpretation of this finding is that underlying factors such as structural racism, poverty, and inequality in society contribute to both medical distrust and depressive symptoms (Budhwani et al., 2015; Patel et al., 2018).

We found medical trust tends to increase with age, consistent with at least one previous study (Mutchler et al., 2019), and over a longer period of living with HIV. Among those more recently diagnosed, medical trust decreases in this early period of HIV diagnosis. Then, after living with HIV for several years, medical trust tends to increase. These findings suggest that PLWH adapt to living with HIV, but that the early stages of doing so are challenging, particularly for young people (Kutnick et al., 2017; Zanoni & Mayer, 2014). Similarly, Kutnick et al. (2017) found that those diagnosed with HIV are not always able to immediately fully accept the diagnosis, acceptance of HIV diagnosis is not necessarily linear, and acceptance of one’s HIV status can sometimes take years. We did not find racial/ethnic differences in patterns of trust, nor was trust related to sex, homelessness, education, or employment, in contrast to past research described above.

We found relationships between depressive symptoms and medical trust vary by age. Among those with elevated depressive symptoms, younger participants have lower levels of medical trust than older participants. Among those without depressive symptoms, medical trust is higher across all ages, and among the oldest PLWH in the sample, medical trust scores are higher than among younger PLWH across all levels of depression. Thus, younger AABL PLWH with depressive symptoms may be most likely to experience this constellation of substantial medical distrust, which can, in turn, impede HIV care continuum engagement. Further, while barriers to treatment for depression are likely multifaceted for this population, past research suggests medical distrust may impede treatment for depression (Nicolaidis et al., 2010). We found PTSD is another important predictor of lower levels of provider and health care system trust, but not a predictor of counter-narrative beliefs. This suggests aspects of mental health in addition to depression might have a negative impact on medical trust. Participants whose sexual orientations are bisexual and “other” report less trust in the healthcare system than those who identify themselves as homosexual/gay/lesbian/queer or heterosexual. Poverty, as indicated by running out of money for necessities more often, predicts less trust in the health care system and greater endorsement of counter-narratives but did not predict trust in providers.

Effective behavioral and/or pharmacological treatments for depression, including among PLWH, exist (Gartlehner et al., 2017), but may be under-used, particularly among AABL PLWH. In a study of PLWH in HIV care settings, almost half demonstrated the need for antidepressant treatment, but AABL PLWH were less likely to initiate antidepressant treatment compared to White PLWH (Bengtson et al., 2016), a finding consistent with other past studies (Sueoka et al., 2010). Among PLWH, a number of behavioral interventions and treatments have successfully decreased depression among PLWH. For example, Shi and colleagues found cognitive behavioral therapy significantly effective on reducing depression at the short-term and reducing viral load at the long-term (Shi et al., 2019). Sherr et al. (2011) found that psychotropic and HIV-specific health psychology interventions were generally effective to decrease depression and to increase ART adherence. Our findings also suggest that interventions aimed at reducing depression may bring multiple benefit beyond enhancing medical trust, such as improving medication adherence and achieving HIV viral suppression. Gaston and Alleyne-Green (2013) provide recommendations for improving trust between AABL PLWH and health care settings and providers. These include the recommendation that providers consider their patients’ past relationships with the health care system, explore their patients’ beliefs about HIV primary care, and also build personal relationships to understand what may motivate their patients to engage in care. They recommend that health care providers examine their own biases and practice-related behaviors (Gaston & Alleyne-Green, 2013). Moreover, culturally salient intervention programs that successfully locate, engage, and treat AABL PLWH have been created and tested (Alegría et al., 2014). Further, distrust has been addressed in social/behavioral interventions. For example, Gwadz and colleagues addressed medical distrust in a peer-driven behavioral intervention designed to increase participation of AABL PLWH in HIV/AIDS clinical trials (Gwadz et al., 2011, 2014; Leonard et al., 2013). The intervention was highly efficacious (Gwadz et al., 2011, 2014), but levels of medical distrust, including counter-narratives, remained static (Gwadz, 2008). Yet, intervention sub-components to actively elicit and understand participants’ distrust and counter-narrative beliefs and experiences, without challenging or correcting such beliefs, are considered critical to this intervention, even if distrust levels and counter-narrative beliefs do not decrease in response to the intervention (Gwadz et al., 2011, 2014; Leonard et al., 2013). Derose et al. (2016) tested an intervention in faith-based settings to reduce HIV stigma and distrust and increase HIV testing. Reductions in distrust were found in Latino but not African American/Black churches (Derose et al., 2016). Thus, in the absence of policy reforms such as programs to eliminate poverty, inequality, and structural racism and community-level stigma reduction, the literature suggests health care settings and providers can improve relationships with patients and better engage them in care by implementing outreach, engagement, and treatment approaches that attend to and explicitly elicit and seek to understand medical distrust beliefs, but without attempting to correct such beliefs, tailored to populations that experience such distrust, consistent with a structural competency approach (Metzl & Hansen, 2014).

### Limitations

The study has some limitations. First, our predictor variables, while plausibly related to distrust based on part literature, explain only 5% of the variation for the overall trust variable in our analysis. This may be due to participants sharing the same social and policy setting, and thus similar social demographic characteristics. We might have observed more variation in trust and more variation accounted for by predictors if our sample had included PLWH who are well-engaged in HIV care and virally suppressed, not in poverty, and not AABL. Further, participants were generally older and long-term survivors of HIV. Results may not generalize to younger AABL PLWH and those outside of service-rich urban settings. Moreover, there are substantial individual differences in trust not predicted by the variables considered. Several important variables are correlated with age (e.g., years since HIV diagnosis), and these variables may be effective only as stand-ins for age when age is not randomly selected for consideration in a particular tree. Last, as a cross-sectional study, we cannot make causal inferences. The fact that medical trust is far from its maximum score and the modest ability of the variables considered to predict it suggested most patients with HIV infection and non-suppressed HIV viral load may benefit from interventions to enhance trust directed at health care settings in particular, and secondarily, health care providers.

### Implications

The present study yields implications for health care settings, clinical practice, future research, and policy. To improve engagement in HIV care and the patient experience, the present study findings highlight the utility of actively eliciting and seeking to understand participants’ distrust, including counter-narrative beliefs. HIV care settings can identify and ameliorate practices and policies that contribute to distrust, and consider distrust as a barrier to care among those with depressive symptoms, including younger people, and those more recently diagnosed with HIV, and with PTSD. Future research is needed to better understand the range of psychosocial factors that contribute to distrust and their interactions, and to identify effective strategies to improve HIV care settings to better engage patients with high levels of distrust. Policies to reduce the larger structural and societal conditions that contribute to distrust, including related to structural racism, have potential to reduce medical distrust and its adverse effects on the health and well-being of AABL PLWH.

## Conclusions

The present study extends and adds nuance to the literature on medical trust/distrust and focuses on an important but understudied subpopulation of PLWH, AABL individuals insufficiently engaged along the HIV care continuum. Findings suggest numerous potential points for intervention to improve HIV care continuum engagement in this population at-risk, and, therefore, will be of interest to HIV care settings, HIV policymakers, and other stakeholders.

## Supplementary Material

Table 4 Trust SUPPLEMENTAL Marginal-Effects

## Figures and Tables

**Table 1.** Sample Description (*N* = 512).

	Mean (SD) or *N* (%)
Age in years	47.0 (10.7)
Sex assigned at birth (male)	359 (70.1%)
African American/Black race (non-Hispanic)	351 (68.6%)
Latino/Hispanic	161 (31.4%)
Gender identity	
Cisgender male	325 (63.5%)
Cisgender female	146 (28.5%)
Transgender	41 (8.0%)
Sexual orientation	
Heterosexual/straight	268 (52.3%)
Homosexual/gay/lesbian/queer/down-low	145 (28.3%)
Bisexual	67 (13.1%)
Other	16 (3.1%)
Don’t know	10 (2.0%)
Refuse to answer	6 (1.2%)
Education (high school/equivalent or higher)	359 (70.1%)
Currently employed (full or part-time)	42 (8.2%)
Incarceration history	
Never	211 (41.2%)
Past, not recent	240 (46.9%)
Recent	52 (10.2%)
History of homelessness	
Currently homeless/unstably housed	211 (41.2%)
Homeless in past year, not currently	56 (10.9%)
Homeless lifetime, not in past year	191 (37.3%)
Never homeless	54 (10.5%)
Indications of extreme poverty	
Less than monthly	220 (42.9%)
Monthly or more	292 (57.1%)
Years since HIV diagnosis	18.2 (8.6)
Received HIV care in the past year	484 (94.7%)
Risk category—Tobacco products	
Lower risk	89 (17.4%)
Moderate risk	346 (67.6%)
High risk	77 (15.0%)
Risk category—Alcohol	
Lower risk	279 (54.5%)
Moderate risk	158 (30.9%)
High risk	75 (14.6%)
Risk category—Cannabis	
Lower risk	191 (37.3%)
Moderate risk	255 (49.8%)
High risk	66 (12.9%)
Highest risk category for other substances	
Lower risk	154 (30.1%)
Moderate risk	243 (47.5%)
High risk	115 (22.5%)
Depressive symptoms (range 0–27)	8.0 (6.4)
Likely PTSD	149 (29.1%)
Outcome variables (POMP scores)	
Trust in providers (range 0–100)	68.7 (19.2)
Trust in health care systems (range 0–100)	56.0 (22.4)
Lack of HIV counter-narratives (range 0–100)	57.3 (21.7)
Combined medical trust variable (range 7.5–100)	60.6 (16.0)

**Table 2.** Variable Importance in the Random Forest Results.

	% MSE increase			
Variable	Counter-narratives	Trust in provider	Trust in healthcare system	Total trust
Depressive symptoms	9.24	4.74	12.89	15.31
Age	10.95	6.56	3.32	7.46
PTSD	−0.97	5.01	10.07	6.51
Running out of money for necessities	1.68	3.31	4.37	4.52
Years since HIV diagnosis	2.66	5.28	1.49	2.11
Gender identity	0.34	0.14	−0.93	1.36
Incarceration history	−0.38	3.11	0.20	1.20
Sexual orientation	1.32	1.26	7.13	1.06
Non-Hispanic black	0.28	−1.17	−0.71	0.92
Homelessness history	0.23	−0.52	−0.83	0.85
Education	−0.91	−1.03	0.08	0.12
Risk category tobacco	6.72	−0.45	2.95	0.09
Highest risk category other substances	−1.65	1.65	1.69	−0.42
Currently working	−1.18	−0.37	0.68	−1.78
Risk category alcohol	−1.90	−2.28	−0.75	−2.67
Risk category cannabis	−1.30	−2.86	−0.95	−2.79

	Number of nodes			
Variable	Counter-narratives	Trust in provider	Trust in healthcare system	Total trust
Age	1,347	1,278	1,158	1,312
Depressive symptoms	1,240	1,148	1,277	1,254
Years since HIV diagnosis	1,039	1,111	1,003	1,037
Running out of money for necessities	924	898	918	936
Sexual orientation	767	816	801	737
Homeless history	625	518	607	592
Incarceration history	382	663	556	536
Education	492	423	479	440
Highest risk category other substances	362	467	418	411
Risk category tobacco	498	388	450	397
Risk category cannabis	375	495	343	387
Gender identity	418	356	302	380
PTSD	204	271	408	319
Risk category alcohol	421	350	356	303
Non-Hispanic black	224	188	239	245
Currently working	182	130	185	214

*Note*. For both indices, a higher value indicates higher relative importance in predicting the medical trust variable.

**Table 3.** Top 10 Interactions in the Random Forest Results.

Counter-narratives		Trust in provider		Trust in healthcare system		Total trust	
Interaction	Occurrences	Interaction	Occurrences	Interaction	Occurrences	Interaction	Occurrences
Depressive symptoms × Age	294	Depressive symptoms × Age	243	Depressive symptoms × Age	289	Depressive symptoms × Age	310
Depressive symptoms × Years since HIV diagnosis	260	Depressive symptoms × Years since HIV diagnosis	236	Depressive symptoms × Extreme poverty	259	Depressive symptoms × Years since HIV diagnosis	279
Age × Depressive symptoms	252	Age × Depressive symptoms	228	Depressive symptoms × Years since HIV diagnosis	254	Depressive symptoms × Extreme poverty	259
Depressive symptoms × Extreme poverty	229	Age × Years since HIV diagnosis	225	Depressive symptoms × Sexual orientation	246	Depressive symptoms × Homeless	239
Depressive symptoms × Sexual orientation	222	Age × Extreme poverty	220	Depressive symptoms × Depressive symptoms	220	Depressive symptoms × Sexual orientation	236
Depressive symptoms × Depressive symptoms	222	Extreme poverty × Age	208	Extreme poverty × Age	203	Age × Depressive symptoms	228
Age × Extreme poverty	216	Age × Age	206	Extreme poverty × Depressive symptoms	200	Depressive symptoms × Depressive symptoms	227
Age × Years since HIV diagnosis	208	Depressive symptoms × Extreme poverty	200	Depressive symptoms × Incarceration	192	Extreme poverty × Age	223
Depressive symptoms × Homeless	207	Age × Sexual orientation	195	Extreme poverty × Years since HIV diagnosis	192	Extreme poverty × Years since HIV diagnosis	214
Age × Age	203	Extreme poverty × Depressive symptoms	192	Depressive symptoms × Homeless	189	Extreme poverty × Depressive symptoms	207

*Note*. An interaction between a variable and itself indicates consecutive splits made on that same variable (e.g., a split at age ≥50 followed by a split at age ≥30 within the <50 branch of the regression tree). This also could be thought of as a nonlinear effect of the predictor (e.g., among those <50 the outcome increases with age, but for those ≥50, no change in the outcome is expected with increasing age).

## Data Availability

The datasets used and/or analyzed during the current study are available from the corresponding author on reasonable request.
