# Supplementary material for: Understanding Medical Distrust Among African American/Black and Latino Persons Living With HIV With Sub-Optimal Engagement Along the HIV Care Continuum: A Machine Learning Approach
Source: Sage Open. Author manuscript; Available in PMC 2022 Jul 7. (PMC9262282; doi:10.1177/21582440211061314)
Supplement: Table 4 Trust SUPPLEMENTAL Marginal-Effects [file NIHMS1819727-supplement-Table_4_Trust_SUPPLEMENTAL_Marginal-Effects.docx]

**Table** **4**

*Marginal Effects*

| Predictors | yhat | | | |
| --- | --- | --- | --- | --- |
|  | Counter Narratives | Provider Trust | Healthcare System Trust | Total Trust |
| Depressive Symptoms |  |  |  |  |
| 0 | 60.94 | 71.20 | 60.05 | 64.14 |
| 1 | 60.98 | 70.29 | 59.64 | 63.88 |
| 2 | 60.27 | 69.27 | 59.50 | 63.35 |
| 3 | 60.34 | 69.57 | 59.33 | 63.38 |
| 4 | 59.10 | 69.30 | 57.58 | 62.30 |
| 5 | 58.57 | 69.10 | 57.14 | 61.86 |
| 6 | 57.80 | 68.67 | 56.31 | 60.68 |
| 7 | 57.23 | 68.50 | 55.56 | 60.16 |
| 8 | 55.62 | 68.14 | 54.94 | 59.27 |
| 9 | 55.29 | 67.40 | 54.66 | 58.84 |
| 10 | 55.16 | 67.30 | 54.48 | 58.64 |
| 11 | 55.65 | 67.20 | 54.77 | 58.85 |
| 12 | 55.48 | 66.89 | 53.68 | 58.42 |
| 13 | 55.48 | 66.79 | 52.81 | 58.10 |
| 14 | 55.32 | 66.60 | 52.45 | 57.94 |
| 15 | 55.19 | 66.61 | 51.95 | 57.78 |
| 16 | 55.16 | 66.62 | 51.84 | 57.79 |
| 17 | 54.84 | 66.81 | 51.78 | 57.78 |
| 18 | 54.81 | 67.20 | 51.83 | 57.80 |
| 19 | 54.79 | 67.30 | 51.70 | 57.83 |
| 20 | 54.78 | 67.18 | 51.61 | 57.75 |
| 21 | 54.85 | 67.78 | 52.02 | 57.84 |
| 22 | 54.82 | 67.79 | 52.22 | 57.82 |
| 23 | 54.69 | 67.78 | 52.83 | 57.94 |
| 24 | 54.37 | 67.77 | 53.03 | 57.77 |
| 25 | 54.80 | 68.04 | 52.97 | 57.76 |
| 26 | 53.29 | 68.40 | 52.95 | 57.74 |
| 27 | 52.66 | 68.40 | 52.95 | 57.72 |
| Age |  |  |  |  |
| 19.00 | 56.51 | 64.12 | 54.59 | 58.30 |
| 20.02 | 56.51 | 64.20 | 54.59 | 58.30 |
| 21.04 | 56.51 | 64.28 | 54.59 | 58.30 |
| 22.07 | 56.03 | 64.28 | 54.59 | 58.26 |
| 23.09 | 55.90 | 64.28 | 54.59 | 58.26 |
| 24.11 | 55.91 | 64.33 | 54.64 | 58.31 |
| 25.13 | 55.68 | 64.44 | 54.61 | 58.30 |
| 26.16 | 55.74 | 64.37 | 54.60 | 58.24 |
| 27.18 | 56.20 | 64.78 | 54.81 | 58.71 |
| 28.20 | 56.22 | 65.21 | 55.15 | 59.08 |
| 29.22 | 56.17 | 65.23 | 55.25 | 59.08 |
| 30.24 | 56.23 | 65.45 | 55.47 | 59.27 |
| 31.27 | 56.36 | 66.02 | 55.43 | 59.48 |
| 32.29 | 56.34 | 66.27 | 55.46 | 59.53 |
| 33.31 | 56.41 | 66.45 | 55.54 | 59.67 |
| 34.33 | 56.46 | 66.38 | 55.54 | 59.70 |
| 35.36 | 56.44 | 66.47 | 55.59 | 59.71 |
| 36.38 | 56.44 | 66.51 | 55.63 | 59.71 |
| 37.40 | 56.45 | 66.65 | 55.68 | 59.77 |
| 38.42 | 56.39 | 66.74 | 55.69 | 59.76 |
| 39.44 | 56.39 | 67.12 | 55.75 | 59.81 |
| 40.47 | 56.36 | 67.07 | 55.76 | 59.81 |
| 41.49 | 56.34 | 66.79 | 55.71 | 59.69 |
| 42.51 | 56.44 | 68.48 | 55.94 | 60.21 |
| 43.53 | 56.57 | 68.52 | 55.95 | 60.35 |
| 44.56 | 56.52 | 68.39 | 55.87 | 60.29 |
| 45.58 | 56.50 | 68.64 | 55.80 | 60.26 |
| 46.60 | 56.52 | 68.61 | 55.78 | 60.26 |
| 47.62 | 56.45 | 68.47 | 55.72 | 60.24 |
| 48.64 | 56.42 | 68.54 | 55.75 | 60.34 |
| 49.67 | 56.38 | 68.94 | 55.77 | 60.36 |
| 50.69 | 56.35 | 69.76 | 55.49 | 60.29 |
| 51.71 | 56.34 | 69.95 | 55.70 | 60.51 |
| 52.73 | 56.44 | 70.00 | 55.83 | 60.80 |
| 53.76 | 57.96 | 70.07 | 56.06 | 61.58 |
| 54.78 | 58.62 | 69.97 | 56.50 | 61.79 |
| 55.80 | 58.99 | 70.03 | 56.66 | 61.88 |
| 56.82 | 58.75 | 69.98 | 56.66 | 61.81 |
| 57.84 | 58.82 | 70.09 | 57.01 | 61.88 |
| 58.87 | 59.30 | 70.07 | 57.76 | 62.12 |
| 59.89 | 59.81 | 69.91 | 57.96 | 62.28 |
| 60.91 | 60.61 | 69.33 | 57.70 | 62.12 |
| 61.93 | 62.89 | 70.07 | 59.43 | 64.36 |
| 62.96 | 63.37 | 70.17 | 59.79 | 64.54 |
| 63.98 | 62.93 | 69.65 | 59.58 | 64.16 |
| 65.00 | 65.25 | 69.67 | 60.05 | 64.47 |
| PTSD |  |  |  |  |
| No | 57.45 | 68.86 | 57.07 | 61.00 |
| Yes | 57.15 | 68.08 | 53.35 | 59.65 |
| Years Since HIV Diagnosis |  |  |  |  |
| 0.00 | 56.50 | 67.20 | 56.20 | 60.39 |
| 0.86 | 56.69 | 66.90 | 56.20 | 60.36 |
| 1.71 | 56.40 | 66.95 | 56.10 | 60.07 |
| 2.57 | 56.28 | 67.02 | 56.02 | 59.91 |
| 3.43 | 56.26 | 67.03 | 56.01 | 59.89 |
| 4.29 | 56.08 | 67.02 | 55.94 | 59.76 |
| 5.14 | 56.15 | 67.29 | 56.01 | 59.86 |
| 6.00 | 56.10 | 67.31 | 56.02 | 59.87 |
| 6.86 | 56.23 | 67.46 | 56.14 | 59.98 |
| 7.71 | 56.27 | 67.54 | 55.90 | 59.98 |
| 8.57 | 56.19 | 67.75 | 55.82 | 59.99 |
| 9.43 | 56.19 | 67.75 | 55.82 | 60.00 |
| 10.29 | 56.22 | 67.80 | 55.64 | 60.00 |
| 11.14 | 56.10 | 67.72 | 55.59 | 59.97 |
| 12.00 | 56.12 | 68.10 | 55.58 | 60.00 |
| 12.86 | 55.94 | 68.06 | 55.56 | 59.97 |
| 13.71 | 55.89 | 68.22 | 55.66 | 60.07 |
| 14.57 | 55.90 | 68.01 | 55.57 | 60.06 |
| 15.43 | 55.95 | 68.02 | 55.56 | 60.07 |
| 16.29 | 56.95 | 68.10 | 55.64 | 60.20 |
| 17.14 | 57.31 | 68.06 | 55.58 | 60.29 |
| 18.00 | 57.49 | 69.47 | 55.57 | 60.42 |
| 18.86 | 57.28 | 68.89 | 55.40 | 60.28 |
| 19.71 | 57.31 | 69.00 | 55.42 | 60.33 |
| 20.57 | 57.37 | 69.16 | 55.42 | 60.49 |
| 21.43 | 57.39 | 69.11 | 55.45 | 60.50 |
| 22.29 | 57.70 | 69.12 | 55.39 | 60.62 |
| 23.14 | 57.84 | 69.16 | 55.58 | 60.69 |
| 24.00 | 57.74 | 69.06 | 55.79 | 60.71 |
| 24.86 | 57.95 | 69.07 | 56.66 | 61.18 |
| 25.71 | 58.06 | 68.75 | 56.78 | 61.12 |
| 26.57 | 58.05 | 68.73 | 56.79 | 61.22 |
| 27.43 | 58.05 | 68.74 | 56.80 | 61.23 |
| 28.29 | 58.12 | 68.81 | 56.83 | 61.30 |
| 29.14 | 58.92 | 68.96 | 57.34 | 61.77 |
| 30.00 | 58.77 | 69.53 | 57.48 | 61.95 |
| Running Out of Money for Necessities | |  |  |  |
| Never | 58.56 | 69.44 | 55.80 | 61.30 |
| Daily | 55.86 | 66.23 | 55.33 | 59.22 |
| Weekly | 55.83 | 66.27 | 54.15 | 58.84 |
| Monthly | 57.04 | 68.37 | 54.75 | 60.06 |
| Occasionally | 57.88 | 69.67 | 57.86 | 61.65 |
| Don’t Know | 58.76 | 69.65 | 57.10 | 62.47 |
| Homelessness History |  |  |  |  |
| Currently Homeless | 56.78 | 68.37 | 55.39 | 60.06 |
| Homeless in Past Year, Not Currently | 56.85 | 68.41 | 56.17 | 60.52 |
| Homeless Lifetime, Not in Past Year | 57.62 | 68.99 | 56.18 | 60.86 |
| Never Homeless | 58.37 | 69.13 | 57.71 | 61.78 |
| Gender identity |  |  |  |  |
| Cisgender Male | 57.42 | 68.75 | 56.26 | 60.73 |
| Cisgender Female | 57.11 | 68.58 | 55.64 | 60.45 |
| Transgender | 56.95 | 68.83 | 55.87 | 60.63 |
| Incarceration history |  |  |  |  |
| Never | 57.28 | 68.88 | 56.40 | 60.74 |
| Past, Not Recent | 57.25 | 69.23 | 56.07 | 60.73 |
| Recent | 57.55 | 66.77 | 54.84 | 60.21 |
| Unavailable | 57.29 | 63.56 | 53.97 | 58.52 |
| Risk Category Tobacco |  |  |  |  |
| High Risk | 56.77 | 68.70 | 56.18 | 60.61 |
| Lower Risk | 59.02 | 68.05 | 57.04 | 61.07 |
| Moderate Risk | 56.94 | 68.87 | 55.71 | 60.52 |
| Sexual Orientation |  |  |  |  |
| Heterosexual/Straight | 57.21 | 69.06 | 56.19 | 60.83 |
| Homosexual/Gay/Lesbian/ Queer/Down-Low | 57.97 | 68.73 | 56.94 | 61.10 |
| Bisexual | 56.21 | 68.59 | 55.05 | 59.83 |
| Other | 56.44 | 65.98 | 52.52 | 58.63 |
| Don’t Know | 57.16 | 65.62 | 51.36 | 58.41 |
| Refuse to Answer | 58.11 | 67.05 | 58.41 | 61.45 |
| Highest Risk Category Other Substances | |  |  |  |
| High Risk | 57.22 | 68.18 | 55.44 | 60.29 |
| Lower Risk | 57.38 | 68.15 | 56.17 | 60.48 |
| Moderate Risk | 57.28 | 69.22 | 56.16 | 60.81 |
| Non-Hispanic Black |  |  |  |  |
| No | 56.86 | 68.42 | 55.50 | 60.14 |
| Yes | 57.54 | 68.89 | 56.33 | 60.90 |
| Currently Working |  |  |  |  |
| No | 57.35 | 68.73 | 56.11 | 60.69 |
| Yes | 57.06 | 68.46 | 55.45 | 60.12 |
| Education |  |  |  |  |
| HS Diploma or GED | 57.86 | 68.82 | 56.43 | 60.86 |
| No HS Diploma/GED | 56.57 | 68.56 | 56.52 | 60.55 |
| Some College or More | 57.39 | 68.81 | 55.14 | 60.47 |
| Risk Category Alcohol |  |  |  |  |
| High Risk | 56.49 | 69.07 | 56.16 | 60.57 |
| Lower Risk | 57.55 | 68.60 | 55.90 | 60.63 |
| Moderate Risk | 57.36 | 68.72 | 56.23 | 60.69 |
| Risk Category Cannabis |  |  |  |  |
| High Risk | 56.81 | 67.36 | 55.95 | 60.10 |
| Lower Risk | 57.83 | 68.75 | 55.90 | 60.78 |
| Moderate Risk | 57.08 | 69.05 | 56.21 | 60.71 |
